# Pseudoaneurysm of the Gastroduodenal Artery: A Rare Complication of Bile Duct Surgery

**DOI:** 10.7759/cureus.53209

**Published:** 2024-01-30

**Authors:** Tariq Bouhout, Ayoub Kharkhach, Abdelbassir Ramdani, Abdelhakim Harouachi, Badr Serji

**Affiliations:** 1 Department of Surgical Oncology, Oncology Hospital of Oujda, Faculty of Medicine and Pharmacy of Oujda, University Mohamed Premier, Oujda, MAR

**Keywords:** vascular ligation, open surgery, digestive hemorrhage, common bile duct, gastroduodenal artery aneurysms

## Abstract

Pseudoaneurysm of the gastroduodenal artery (GDA) is an exceptional complication of common bile duct (CBD) resection. We present the case of a 60-year-old woman with a history of cholecystectomy. The patient was admitted to our hospital for surgical management of the cystic dilatation of the CBD. The patient presented on postoperative day 21 with hemodynamic instability related to a pseudoaneurysm of the GDA. An urgent open surgery was performed with dissection and ligation of the GDA.

## Introduction

Gastroduodenal artery (GDA) pseudoaneurysm is a rare vascular lesion that represents less than 1.5% of all splanchnic artery aneurysms [1]. A pseudoaneurysm of the GDA is an exceptional complication of common bile duct (CBD) resection. Herein, we describe an unusual complication resection of the CBD with Roux-en-Y hepaticojejunostomy through a case of a GDA pseudoaneurysm revealed by an upper digestive hemorrhage treated with ligation of the inflow and outflow and the resection of the aneurysm. We reviewed the possible causes, manifestations, diagnostic tools, and treatment alternatives for this situation.

## Case presentation

A 60-year-old female patient with a history of cholecystectomy 17 years ago was admitted to our department for cystic dilatation of the CBD. She had a resection of the CBD with a Roux-en-Y hepaticojejunostomy. The early postoperative course was simple. Her past medical history did not reveal any cancer in her family, smoking, or alcoholism.

The patient presented to the emergency room on postoperative day 21, complaining of abdominal pain extending to the upper quadrants with hematemesis that started five hours before her admission. A physical examination revealed epigastric abdominal tenderness without signs of peritonitis. Laboratory tests showed microcytic normochromic anemia at 7.3 g/dl; besides, liver biochemical tests and routine blood examinations were all within normal limits.

**Figure 1:**
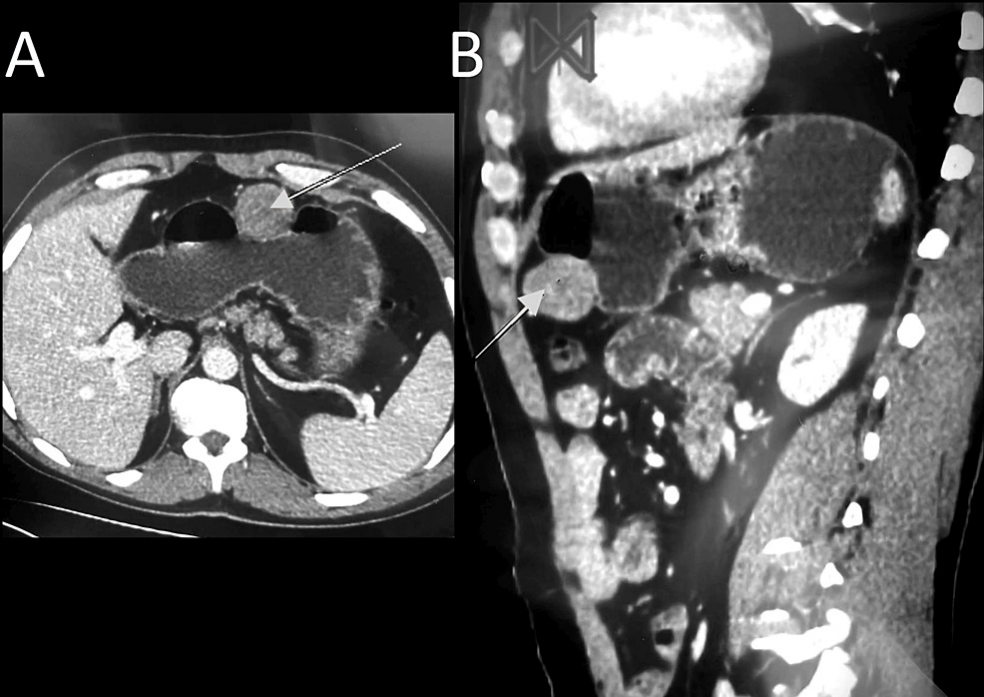
Abdominal CT scan transverse plane (A) and sagittal plane (B) showing homogenous and enhanced vascular mass (arrows) that arose from a splanchnic branch, it was adjacent to the anastomosis performed before, suggesting a pseudoaneurysm of the GDA.

Abdominopelvic computed tomography (CT) scan revealed a homogenous and enhanced vascular mass that arose from a splanchnic branch; it was adjacent to the anastomosis performed before, suggesting a pseudoaneurysm of the GDA (Figure 1A, 1B).

**Figure 2:**
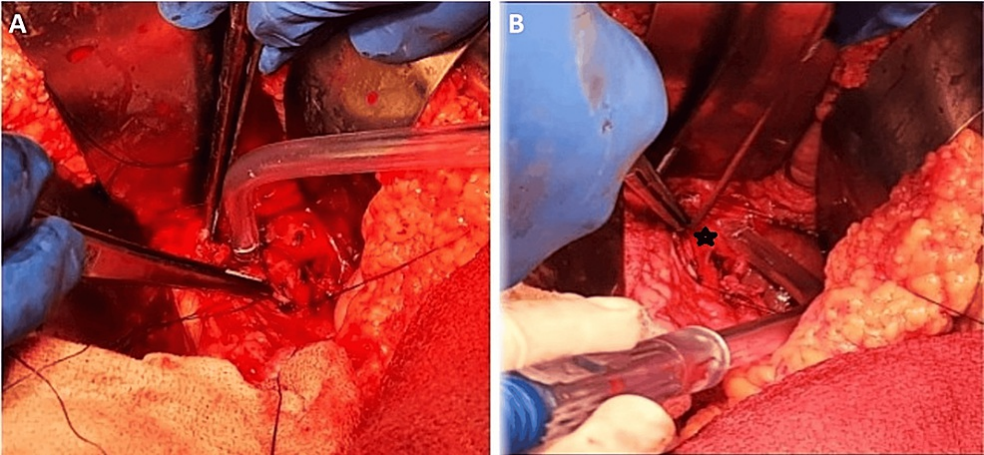
(A) Preoperative view showing a stigmata of bleeding near to hepaticojejunostomy; (B) identification of the pseudoaneurysm (star) and ligation of the inflow and outflow of GDA.

Faced with these clinical and radiological findings and the unavailability of interventional radiologists in our structure, the decision was to proceed with an emergency laparotomy. The patient underwent an upper median laparotomy, and we found stigmata of previous bleeding covering the hepaticojejunostomy with active bleeding around the common hepatic artery (Figure 2A, 2B). GDA pseudoaneurysm was confirmed, and we performed a careful dissection of the GDA with ligation of the inflow and outflow and the resection of the aneurysm.

After an uneventful postoperative course, the patient was discharged on postoperative day 12, with a CT scan scheduled two weeks later. After one year of follow-up, the patient was doing well, and the control CT scan did not show any abnormal signs or recurrence.

## Discussion

Visceral artery aneurysms are very rare (incidence of 0.01-0.2%) and tend to be asymptomatic [2-4]. They concern the celiac trunk, or superior mesenteric artery, and its branches, especially the splenic artery [5]. However, the GDA aneurysm is unusual, with an incidence of 0.01-1% of all visceral artery aneurysms; they result clinically in abdominal pain and haematemesis, with a high risk of rupture [4,6].

It is worthy to distinguish true aneurysms related to vessel abnormalities from pseudoaneurysms or false aneurysms caused by vascular injury to one or more layers of the arterial wall [7,8]. The most common cause of GDA pseudoaneurysm is acute pancreatitis (6-9.5% of patients), followed by infection, inflammation, trauma, and iatrogenic lesions [7-9]. In our case, we assumed that a possible dehiscence of biliodigestive anastomosis was the cause of the erosion of the GDA.

Clinical symptoms of GDA pseudoaneurysm can range from an accidental discovery on a CT scan up to a life-threatening hemorrhage due to a ruptured pseudoaneurysm in 52% of cases [9,10]. The most common symptoms are acute abdominal pain (46%) [9,11]. Haematemesis and meleana may be present if the pseudoaneurysm communicates with a digestive structure [9,10]. Other symptoms might be related to pressure on adjacent organs, such as vomiting and cholestasis [1,4]. Depending on the clinical presentation, upper gastrointestinal endoscopy, CT scan, or angiography will be performed, although the CT scan remains the gold standard for early diagnosis and treatment. However, angiography has a better sensitivity (100% versus 67% for a CT scan) and could be diagnostic and therapeutic at the same time [4,11].

Indeed, if we take into consideration the high risk of rupture of the GDA pseudoaneurysm, with a mortality rate that ranges from 21 to 76% [1,3,12], this situation requires proactive treatment once the diagnosis has been made [4,9,13].

The management of pseudoaneurysms is open to several approaches. We think that the choice of the therapeutic procedure is made on a case-by-case basis without delaying the treatment and according to the available technical platform [3]. Currently, a less invasive procedure is preferred for stable patients; thus, endovascular embolization is a first-line therapy. It has the potential to be employed in patients at higher surgical risk or in locations where aneurysms are difficult to surgically access [1,14]. Furthermore, endovascular treatment has less mortality and better cost-effectiveness with a shorter hospital stay [1,9]

If embolization fails, it is not available, or if patients are unstable, an open surgical approach is recommended, as discussed in our case report. Pseudoaneurysm resection, partial and total gastrectomy, and pancreatectomy are all procedures that can be used [4,9]. Further, successful treatment with percutaneous or endoscopic ultrasound-guided thrombin injection has been documented recently [7,15].

## Conclusions

GDA pseudoaneurysm is an unusual and challenging situation; it should be considered in patients with abdominal pain and gastrointestinal bleeding. Its rupture is a fatal situation. There are many treatment options for endovascular and open-surgical repair. Here, we describe the successful surgical management of a GDA pseudoaneurysm, but the conservative approach with vascular embolization remains the gold standard when available.
